# Use of the term “landscape” in sustainable agriculture research: A literature review

**DOI:** 10.1016/j.heliyon.2023.e22173

**Published:** 2023-11-10

**Authors:** Anna Pereponova, Gunnar Lischeid, Kathrin Grahmann, Sonoko Dorothea Bellingrath-Kimura, Frank A. Ewert

**Affiliations:** a Postdoctoral Researcher at Leibniz Centre for Agricultural Landscape Research (ZALF), Germany; b Co-Head of Research Platform Data Analysis and Simulation at the Leibniz Centre for Agricultural Landscape Research (ZALF, and Professor at University of Potsdam, Institute of Environmental Science and Geography, Germany; c Tenure Track Candidate at the Leibniz Centre for Agricultural Landscape Research (ZALF), Germany; d Co-Head of Research Area 2 at Leibniz Centre for Agricultural Landscape Research (ZALF) and Professor at Humboldt University of Berlin, Department of Agronomy and Crop Science, Germany; e Scientific Director of the Leibniz Centre for Agricultural Landscape Research (ZALF) and Professor at the University of Bonn, Institute of Crop Science and Resource Conservation (INRES), Germany

**Keywords:** Landscape scale, Sustainability, Interdisciplinary research, Transdisciplinarity, Agroecosystems

## Abstract

Finding consensus in definitions of commonly-used terms and concepts is a key requirement to enable cooperations between interdisciplinary scientists and practitioners in inter- or transdisciplinary projects. In research on sustainable agriculture, the term ‘landscape’ is emphasised in particular, being used in studies that range from biogeochemical to socio-economic topics. However, it is normally used in a rather unspecific manner. Moreover, different disciplines assign deviating meanings to this term, which impedes interdisciplinary understanding and synthesis. To close this gap, a systematic literature review from relevant disciplines was conducted to identify a common understanding of the term “landscape”. Three general categories of landscape conceptualizations were identified. In a small subset of studies, “landscape” is defined by area size or by natural or anthropogenic borders. The majority of reviewed papers, though, define landscapes as sets of relationships between various elements. Selection of respective elements differed widely depending on research objects. Based on these findings, a new definition of landscape is proposed, which can be operationalized by interdisciplinary researchers to define a common study object and which allows for sufficient flexibility depending on specific research questions. It also avoids over-emphasis on specific spatio-temporal relations at the “landscape scale”, which may be context-dependent. Agricultural landscape research demands for study-specific definitions which should be meticulously provided in the future.

## 1 Introduction

Agroecosystems cover a quarter of the global terrestrial area, and the ecosystem services (ESS) they provide are extremely important for human society [1]. Demands for ESS increase with the growing standards of life [2], and population, which has quadrupled in the last century [3]. In the last decades, high agricultural production has been supported mainly through intensification, resulting in simplified, high-input arable systems [4]. This adversely affects the ecological functions of agroecosystems, leading to a loss of biodiversity and increasing environmental problems [[4], [5], [6]], making agriculture the key driver of current planetary changes, while it is in turn strongly affected by these changes [7].

In contrast, sustainable agriculture aims at environmentally friendly, socially fair and economically viable food production [8]. Altieri [9] describes agricultural sustainability as “the ability of an agroecosystem to maintain production through time, in the face of long-term ecological constraints and socioeconomic pressures” and stresses the need for understanding coevolution and interdependencies between farmers and their knowledge within their biophysical and environmental conditions. The vision of sustainable agriculture as a dynamic process is also highlighted by FAO [91], which emphasizes its principles such as efficiency of resource use, protection of natural resources and of rural livelihoods, social well-being, resilience of people, communities and ecosystems, and good governance.

A plethora of scientific studies exist, which aim at contributing to sustainability goals with widely differing levels of integration of different anthropogenic activities and effects on the environment. Different types of sustainable agriculture approaches have evolved, such as *organic agriculture* [10], *ecological intensification* [11] and *sustainable intensification* [6,7,12] as well as *agroecology* [11] among others. Most of these approaches acknowledge the demand for a system's inter- or transdisciplinary vision to a varying degree, considering different spatial and temporal scales [11]. However, consensus in defining critical terms and concepts has not yet been achieved sufficiently.

The terms “landscape approach” or “landscape”, which are often used in respect to spatial scale are not clearly defined [[13], [14], [15], [16]]. The multitude of dimensions in which “landscape” is defined has been well demonstrated by Gailing and Leibenath [16] through pairs of contrasting properties, characterising such definitions. These properties manifest to a different degree depending on the context in which this term is used [16], and the definition of landscape depends on the study object. Sayer et al. [13] forwarded a similar idea noting that the term “landscape approach” has a “constructively ambiguous meaning that people can agree on […] in principle while disagreeing on many key details”, and the same can be applied to *landscape*.

While several aforementioned works have systemized landscape definitions from different angles (e.g. [16,17]), a universal and practical cross-disciplinary interpretation is missing. Thus, the objective of this article is to address the practical need of inter- and transdisciplinary researchers to come to a common understanding of landscape, which would also have functional relevance to enable collaboration for transition towards sustainable agriculture. These goals are approached by trying to answer the following research questions:

1) How is the concept of landscape defined in existing scientific literature in relation to agricultural systems?
2) What would be a useful definition of the term “landscape” as an important component and study unit in integrated and inter-/transdisciplinary research on sustainable agriculture?

To achieve this goal, the meaning of “landscape” in recent literature has been analysed. Based on the results, we propose a new definition of landscape as an essential facet for conducting research in the context of sustainable agriculture.

## 2 Evolution of landscape concepts

The word ‘landscape’ is assumed to have originated in Western Europe, specifically from Germanic languages [15,18]. The term entered the English language through the German word ‘Landschaft’ (‘Land’ = ‘a bordered territory’, ‘schaffen’ = to make [15,18]. Kühne [19] adds that ‘Land’ in German has four meanings: a state or legal territory, buildable surface of the earth, the mainland, or the countryside (as opposed to the city). Antrop [15] refers to the Dutch word ‘lantscap’ from the 13th century meaning a land region or environment, while an earlier word ‘Lantscaf’ from old German dates back to the 9th century, meaning something ‘that has the quality of a larger settlement area’ [20], which included the local behavioural society characteristics, but not the exact spatial delimitation, and in the 12th century obtained a component of politically defined space [19]. Cosgrove [18] points to emergence of Landscape as a ‘term, an idea, or better still, a way of seeing the external world’, originating from renaissance humanism, with the appearance of the first painting and pictures of landscape, which some authors call ‘emergence of landscape conscience’ [14]. The author showed that the further historical development of the landscape concept is connected to the practical appropriation of space and highlights its connection to the history of science and relation to practical activities such as surveying, mapping, and charting. Antrop [15] specified that more systematic landscape exploration began during the Age of Discovery, with advancements in cartography and growing interest from naturalists. In the late 19th century Alexander von Humboldt pointed to the holistic nature and aesthetic qualities of landscapes, and Vidal de la Blache also highlighted the role of local societies in shaping them. In the beginning of the 20th century the concept of landscape was brought into the Anglophonic geographical discussions by Carl Sauer [15], who saw geography as a systematic study on the production and modification of landscapes [21], and where landscape studies adopted qualitative and quantitative methods [21].

In the middle of the 20^th^ century, Carl Troll [22] integrated aerial photography into landscape observation and coined the term ‘landscape ecology’, which promoted holistic synthesis in landscape research [14,15]. In the 1960s and 1970s, a deductive and rationalistic approach dominated geography, but interest in the visual appearance and aesthetics of landscapes was later restored [15]. The first attempt to restore the interdisciplinary field of landscape research was made in the Netherlands in 1972 with the creation of the working group ‘Landscape ecological research’. After the Association of the International Landscape Ecology was founded in 1988, landscape research expanded over many different disciplines, each taking up different shades of its meaning [15,21].

In parallel, the ‘cultural landscape’ concept was proposed by F. Ratzel (in 1895–1896) on the rise of human geography, while O. Schluter (in 1906) specified its relevance to human society through ‘cultural landscape theory’ [23,24]. Cultural, including agricultural, landscapes received wider acceptance in the 20^th^ century through increased academic research and were recognized under the World Heritage Convention in 1992, leading to even more studies around them [[23], [24], [25]]. Hence, many concepts of agricultural landscape appeared in the last decades [26]. While in 1990 Meeus et al. [27] defined them as ‘landscapes used for agricultural purposes, that are managed to provide adequate and long-term production capacity’‘, the definition by the Organisation for Economic Co-operation and Development in 2001 [28] -’‘ … the visible outcomes resulting from the interaction between agricultural commodity production, natural resources and the environment’‘- emphasizes non-production values, and considers them as ‘socio-economic ecosystems’.

In 2010, Kruse et al. [29] proposed a glossary of agricultural landscapes to serve as a common basis, where they define agricultural landscapes as ‘‘landscapes which are strongly related to past and/or present agricultural activity’‘, which may contain farmland, cultivated land, grasslands, meadows; horticulture, viticulture, olive or trees; small infrastructure elements; non-crop vegetation structures; forest mosaic and elements within an agricultural context (both spatial and functional). However, in a study by Andersen [26], he still identified seven groups of agricultural landscape concepts found in publications in the European Journal of Agronomy since 2010. He proposes his own concept of agricultural landscape *‘as a distinct pattern of farming systems and landscape elements in a homogeneous biophysical and administrative endowment’*, providing a generic way of how it can be operationalized, and specifying both a conceptual and a quantitative landscape approach that can support farming design at the landscape level. However, this general definition takes into account a limited number of landscape components in a generic way and focuses mainly on spatial patterns of farming systems. Although this approach is practical for characterizing or classifying agricultural landscapes based on pre-selected indicators, it does not serve the same purposes as those discussed in this article.

## 3 Methodology

**Fig. 1:**
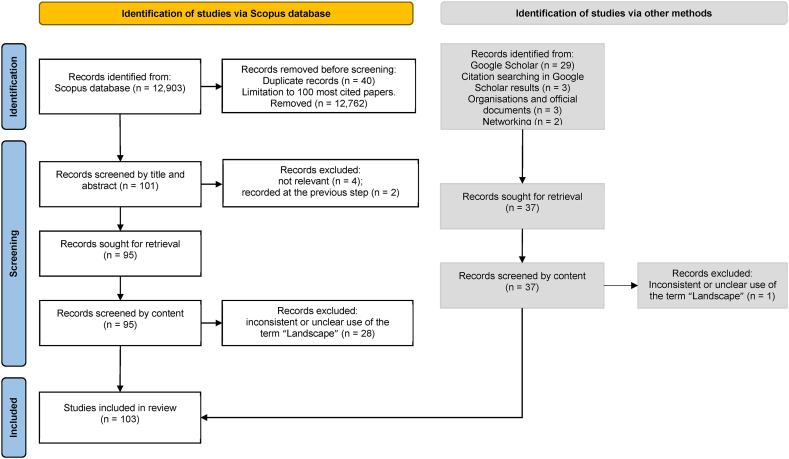
Flow diagram depicting the approach for a systematic review of “Landscape” conceptualization.

A systematic review (following [30,31]) was performed to understand how the term “landscape” is used in the scientific literature in relation to objectives or questions of each particular study. While computer-assisted methodologies of bibliometric analyses for conducting systematic reviews have become available in recent years (e.g. [32,33]), in this study a manual review was preferred, since the context of each individual paper was of specific attention. The definitions or the interpretations of the term “landscape” in scientific articles, conference papers and book chapters on various topics in the context of agriculture and environment were collected and analysed. The protocol was not registered for this review. The workflow is illustrated by a flowchart in Fig. 1, created following Page et al. [34].

As the first step, in order to gain a first, general idea on the interpretations of the landscape concept, a search was conducted in GoogleScholar on the use of Landscape (scale) in agricultural or environmental contexts. This database was first selected, as it covers a wider variety of the types of publications, including non-journal. The search was done using the search words “landscape”, “scale” and “agriculture”. The latter two keywords were added in an attempt to narrow down the amount of results for such a broad criteria by stressing the relevance to scale issues as in the interest of this article. The search was not limited by the date of publication, since it was considered important to get an overview of interpretations of landscape over a longer time period. No other filters were set, due to limitations of functionality of GoogleScholar. Out of the two options of sorting the results available in GoogleScholar (by relevance or date), the first was selected as more suitable. Due to the large number of results returned for this broad search definition, the first four pages from the list of articles (40 research items) were scanned by titles and abstracts in search for papers with relevant content. This search was conducted on 3^rd^ January 2022 and resulted in 29 studies, for which the full-texts were further searched for and screened. It should be noted that not only the works with explicit definitions were targeted, but also the ones which provide a clear understanding and vision of landscape interpretation. Three more research items were added through cross-conferences found in these studies, and, in a similar manner, two official definitions by relevant international organizations (UNESCO [89]; IUCN [88]) and an official document (Landscape Convention: Council of Europe [90]) were identified and added to these results. One article was excluded after reading the full-text, as the meaning of “landscape” from its text could not be deducted unequivocally. This resulted in a list of 34 studies. Additionally, interdisciplinary experts were contacted for advice of popular opinions in their field, and two more publications, which did not appear in the list were added based on their recommendations. Based on findings from these last three groups of results (“other” methods in Fig. 1), three main groups of conceptualization approaches of “landscape” were identified (details are provided in the subchapter 4.1 Categories of Landscape concepts).

As the second step, an additional search in Scopus database was used to further refine this categorization and demonstrate which of them were more or less common specifically in peer-reviewed literature in the relevant scientific fields. Scopus was selected in this case, since it accommodates the selection of scientific disciplines which are relevant to the current discussion, allows to target peer-reviewed scientific literature, as in the interest of this paper, and to indicate detailed search criteria. In comparison to Web of Science, as another major database which also covers the scientific fields of our interest, Scopus covers more publications and includes most of the documents of the article type [35]. Specifically, it was previously shown that almost all the publications with higher citation rate in Web of Science are also present in Scopus) [35]. A relatively broad choice of keywords had to be used for the search in Scopus as well, which included the search words ‘Landscape’ (in title and abstract), and ‘Agri’ OR ‘Agro (in all text elements). The search was limited by research areas to agricultural (‘AGRI’) and environmental (‘ENVI’) research fields, to English language, and by the publication stage to ‘Final’. The search in Scopus database was conducted on February 18, 2022 and was not limited by the period (years) in which the studies were published. The search results (12,903) were downloaded as a CSV file with basic information on the articles. After removing duplicates (40) within this list, a total of 12,864 items published between 1976 and 2022 were identified. As it was deemed important to get an overview of the most common ways of definition, but such a large number of findings would have been unfeasible for manual review, it was decided to select the 100 most popular papers, where the citation rate was used as a proxy for popularity. An additional, 101^st^, article was included, due to having an equal number of citations to the least cited paper in the selection. Next, the full texts of these 101 selected papers were retrieved and scanned for relevance by titles and abstracts. During the scanning step, four papers were excluded due to irrelevant topics, and two more papers were excluded as they had already been recorded at the previous step. After reading through the full texts of the remaining 95 studies, 28 of them were excluded, due to inconsistent or unclear use of the term “landscape”. The remaining 67 studies were merged with the results of the first step, and the combined data were organized in a table, categorizing them based on research topics and conceptualization approaches. The screening of the records was done manually by the same reviewer for both steps. This was done to avoid inconsistencies in judgements. The risk of reporting bias was deemed negligible due to the clearly defined selection strategy. The results were, however, further discussed with the co-authors. Though the risk of publication bias is usually attributed to selection of highly-cited papers, it was not applicable in this case, since the goal was to select the most popular opinions instead of fully covering all publications, as explained earlier in this section.

In addition to the analysis described above, the historical trend of using the world “landscape” (in title and abstract) AND “agri” OR “agro” (in text) in publications found in Scopus, and published between 1976 and 2021, was created.

Using Google Scholar and additional sources did not allow to preselect the research areas, however, it allowed to get more encompassing overview of existing conceptualizations. Meanwhile, the further search in Scopus has helped to target the research areas under interest more specifically. Table 1 reflects all the publications, which were included for the analysis, sorted according to research topics, and the way landscape is interpreted. The studies, which addressed or mentioned several conceptualizations of landscape (e.g., seminal works on landscape ecology), were included in all relevant categories.

**Table 1:** Distribution of the number of collected publications on landscape conceptualization approaches and their groups (among them 3 studies illustrated both category 3.1 and 3.3, or in 3.1 and 3.6). Here, Social sciences and Environmental sciences refer to “more than a single of sub-discipline” thereof. Please see Supplementary material 1 for the references used in the table.

Category		Conceptualization	Discipline	Research topic	№ of articles	References
Main	Sub					
1	–	Arbitrarily defined area (size)	Ecology	Species abundance and diversity	4	*1, 2, 3, 4*
2	–	Arbitrarily or naturally defined borders	Ecology	Species abundances	1	*5*
			Soil science	Soil health	2	*6, 7*
			Biogeochemistry	Biogeochemical processes	2	*8, 103*
3	3.1	Defined by Visual Perception	Agronomy, Social and Environmental sciences	Multiple ecosystem services	3	*9, 10, 11*
			Landscape ecology	Fundamental works on landscape ecology and theory	2	*12, 13*
			Landscape architecture and planning	Cultural perspective	2	*14, 15*
				Sustainability (Official document)	1	*102*
	3.2	Area of district farming systems and practices	Social and Environmental sciences	LULC change	1	*16*
			Agronomy, Social and Environmental sciences	Agricultural land management	1	*18*
				Adaptive capacity	1	*19*
			UNESCO	Cultural perspective	1	*101*
	3.3	Land Use and Land Cover mosaics within and between farms	Ecology	Species abundance and diversity	34	*20, 21, 22, 23, 24, 25, 26, 27, 28, 29, 30, 31, 32, 33, 34, 35, 36, 37, 38, 39, 40, 41, 42, 43, 44, 45, 46, 47, 48, 49, 50, 51, 52, 53*
			Agronomy	Farm systems evolution and typology	1	*54*
			Ecology and Agronomy	Pest dispersal	1	*55*
			Soil biology	Role of invertebrates for Ecosystem services	1	*56*
			Ecology, Geography and Social sciences	Management -economic perspective	2	*57, 58*
			Forestry, (Landscape) Ecology, Geography, Agronomy	(Wild)fire impact and control	2	*59, 60*
			Agronomy, Social and Environmental sciences	Management of multiple ecosystem services	5	*61, 9, 10, 62, 63*
			Agronomy and Environmental sciences		2	*64, 65*
			Landscape ecology, Ecology		2	*66, 67*
			Social and Environmental sciences	*Greenhouse gas assessment*	1	*68*
			Landscape ecology or geography	*Monitoring/assesment of LULC change - Remote sensing and modelling*	2	*69, 70*
			Conservation Ecology		1	*71*
			Landscape ecology	Fundamental works on landscape ecology and theory	2	*13, 72*
	3.4	“Spatial arrangement of ecological resources"	Biogeochemistry	Biogeochemical processes	2	*73, 74*
			Geological science	Geomorphology aspects	2	*75, 76*
			Agronomy and Environmental Sciences	Sustainable land management	1	*77*
			Environmental Sciences	Environmental monitoring	2	*78, 79*
			Agronomy, Environmental and social sciences	Soil erosion	1	*80*
	3.5	Spatial arrangement of natural and human-made objects	Hydrology and Ecology	Hydrological connectivity	1	*81*
			Ecology	Invasive species	1	*82*
	3.6	Interrelated human and nature factors and processes and providing various ESS	Social and Environmental sciences	Landscape sustainability and resource management	9	*83, 84, 85, 86, 87, 88, 99, 89, 100*
				LULC change analysis	6	*90, 91, 92, 93, 94, 95*
			Ecology	Biodiversity	1	*96*
			Humanities	Conceptual theoretical discourse	1	*97*
			Agronomy and Environmental Sciences		2	*11, 98*

## 4 Results and discussion

### 4.1 Categories of landscape concepts

**Fig. 2:**
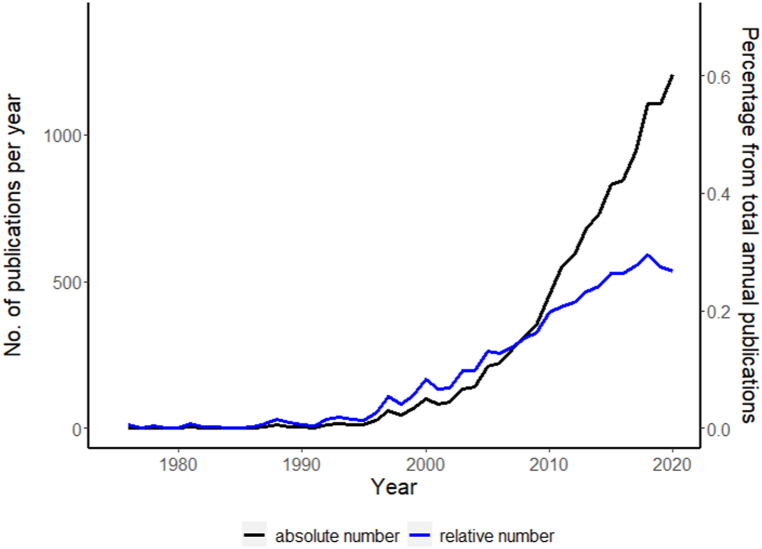
The number of publications listed in the Scopus database published between 1976 and 2021 with the words “landscape” (in title and abstract) AND “agri” OR “agro” (in text) (black line, left axis) and the total number of papers divided by the total number of papers published in agricultural and environmental research fields (blue line, right axis).

The Scopus overview revealed a growing number of publications using “landscape” in the abstract or title (Fig. 2). The increase of the absolute number of publications using this term (black line) could be partly attributed to the general growth of peer reviewed literature in this period. Nevertheless, the relative amounts of such studies (normalized by the total amounts of all peer-reviewed publications in agricultural and environmental research fields in each year) still shows a considerable increase. It can be seen that already by the late 1990s its usage started to steadily accelerate, which reflects the historical trends described in the “Evolution of the Landscape concept” chapter, and highlights the need to systemize our knowledge upon the different perceptions of this term.

**Fig. 3:**
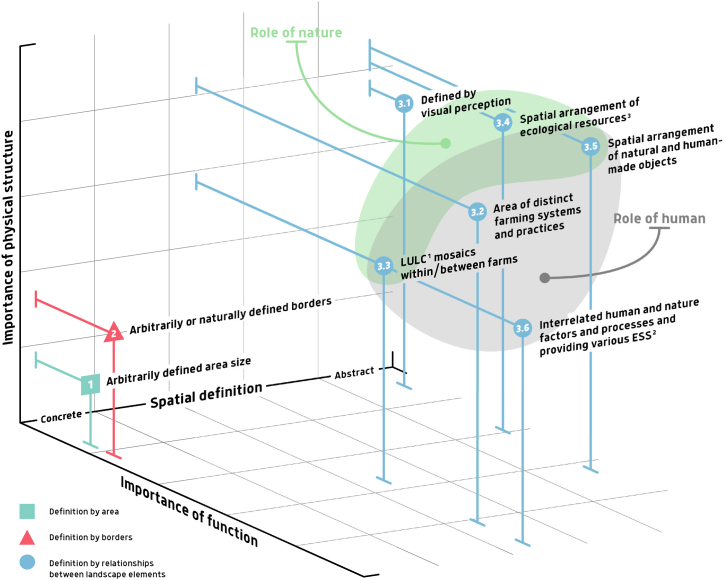
Schematic distribution of collected publications by landscape conceptualization approaches and groups. The reader is referred to Table 1 for additional details. 1LULC = land use and land cover; 2ESS = ecosystem services; 3 O'Neal et al. [36].

The reviewed studies can be divided in three general categories of landscape definitions, as illustrated by Fig. 3 in the dimensions of importance of physical structure or function and the role of human-nature relationships. The 1^st^ category defined landscape by arbitrarily selected size of an area taken for analysis. The 2^nd^ category defined landscape by arbitrarily or naturally defined borders, from smaller administrative units to complete land masses.

The 3^rd^ category focuses on internal relationships between various landscape elements, thus, landscapes are defined here based on internal structure. This category can be divided into subcategories, where landscape is defined:

3.1 through visual perception,

3.2 as an area of distinct farming systems and practices,

3.3 through land use and land cover (LULC) mosaics within or between farms,

3.4 through a spatial arrangement of ecological resources,

3.5 through a spatial arrangement of natural and human-made objects or.

3.6 by interrelated human and nature factors and processes and providing various ESS.

Definition of “landscape” *by arbitrarily selected area size* (category 1 in Fig. 3 and Table 1) was encountered rarely. Examples were found in studies on species abundance and diversity, measured per unit of area (e.g. [37]). There was no consistency in terms of specific area sizes between these studies. In fact, they varied between 1 and 28 km^2^. The sizes were assigned rather arbitrarily to define a part of land suitable for each particular analysis. Though the authors usually did not explain the choice, the landscape was seen rather as a spatial unit, compared or opposed to either field [37], field and farm [5] or “local” scale [38], while the meaning of the latter was typically not specifically explained. These studies recognize the importance of landscape heterogeneity, consisting of composition (“the number and proportions of different cover types”) and configuration (“the spatial arrangement of cover types”) [39], and landscape heterogeneity (in the sense of land cover). However, they opt for a rather formal approach to delineate “landscape” by borders instead of functional or structural attributes.

Defining landscape by *arbitrarily or naturally defined borders* (category 2) (e.g., administrative or borders of a specific LULC category) may indirectly consider the functional attributes of landscape definition, implied similarities in management approaches or socio-political circumstances of surrounding areas. However, such considerations are not usually made explicit and rather represent a comfortable unit of measurement in the studies within this group [40]. A minor part of the studies in this selection use the word “landscape” referring to a particular land use and cover category (e.g. [41]) or to land surface as a whole (i.e. as opposed to oceans) (e.g. [42]).

An intuitive understanding of landscape, which may have appeared the earliest (as mentioned, e.g., by Sayer et al. [13], Penko Seidl et al. [43], Selman [44] and Turner and Gardner [45]) is based on *visual perception* (subcategory 3.1). Here landscape is viewed as a visually distinct, ornamental or spiritually notable place. Though mainly defined by its physical appearance, this landscape conceptualization considers its role in providing a diversity of ESS. Out of those cultural ESS are the most evident, but it is argued that provisioning, supporting and regulatory ESS also play a certain role here, as people intuitively relate landscape appearance to these services [44]. The classical interpretation of this conceptualization has been commonly used to define cultural landscapes, focusing on the influence of human activities in shaping or altering the Earth's surface [23]. Specifically, the term “agricultural landscape” emerges from the dynamic interplay between the region's “geomorphological” and “biotic” characteristics or potentials, along with its anthropogenic use with a very broad concept of use over time. Next to it, landscape as an “*area of district farming systems and practices*” (the subcategory 3.2), though also characterised by visual distinctiveness, has stronger emphasis on functionality and necessarily represents a result of human culture. Still, in this sense “landscape” has a rather nominative meaning (e.g., terrace landscapes), and human-nature relationship is mostly monodirectional.

In most studies landscape was referred to through *spatial organisation or structure and relationship* between its elements. From the results of our review in the agricultural context, landscape is regarded most often as the mosaic (composition and configuration) of LULC (subcategory 3.3). This conceptualization was observed in the majority of the collected publications and is represented largely by ecological studies on species abundance and diversity, at scales from crop fields to clusters of farms and surrounding areas (e.g. [[46], [47], [48], [49], [50], [51], [52]]). Widely used definitions for landscape in this sense describe it as “a kilometres-wide mosaic over which local ecosystems *recur*” [53], or as composed of habitat or non-habitat following the ‘island-mainland’ paradigm for terrestrial landscapes in studies on species abundance or diversity (e.g. [54,55]). Here, landscape is perceived as spatially scalable, spanning vast distances of up to hundreds of kilometers, to encompass short-term dispersal ranges of the majority of organisms [54,56]. Baker [57] describes landscape through a variety of spatially arranged “landscape elements”, stressing that although no lower physical limit exists to this scale, it is measured by an extent of kilometres rather than hundreds of metres. Other studies focus on landscape management from different perspectives of sustainability (e.g. [8]). This category also includes studies on various applications of remote sensing and GIS techniques, such as their potential for large-area conservation monitoring [58], analysis of cause-effect relationships between vegetation fire and vegetation cover and land use [59], or comparison of advanced data analysis methods for classification of agricultural landscapes [60]. More generally, from this functional perspective, landscapes can probably be seen as areas, homogeneous in their heterogeneity, where, as mentioned by Fahrig et al. [39]. The latter should be measured according to the expected functions. In a broader sense, landscape in this case is defined as “spatial arrangement of ecological resources” [36] (subcategory 3.4), referring to the landscape as “two- or three-dimensional” [61]. Examples include, e.g., studies on nutrient fluxes, biosphere–hydrosphere–atmosphere exchange processes and disciplines such as biogeochemistry, geomorphology, studies on soil erosion and landscape management (e.g. [36,62,63]). In a broader sense, landscape as *spatial arrangement of natural and human-made objects* (subcategory 3.5) integrates the effects of objects of human civilization (such as built-up infrastructure), allowing to address, e.g., the anthropogenic impact on landscape connectivity, which can be seen for some examples from ecology or hydrology [64].

A more complex vision of landscape, which is also usually based on the relationship between its elements rather than on borders, considers it as a holistic system built on complex society-ecological interactions (e.g., [13,44; IUCN, 2019]) (subcategory 3.6). In this case, a landscape is defined by merged human and nature processes, where pursuing sustainable development requires balancing social, economic and ecological perspectives [44]. Thus, sustainable development constitutes a product of both, biophysical properties (e.g., topography and productivity of land) as well as of the historical and socioeconomic context of landscape (affecting, e.g., its spatial structure and choices of farming approaches [65]). Studies on natural resources management (e.g. [66]) or climate-smart landscapes [67] can serve as examples. From this point of view, the term ‘landscape’ is predominantly defined functionally, where importance lies in the actual unit areas managed by stakeholders to achieve biodiversity, production and livelihood goals [68] and can be seen through the multifunctionality paradigm (see [69] for the details). However, Gailing and Leibenath [16] mention that the holistic “gestalt” of the landscape concept, while being interdisciplinary and multidimensional, has been criticised for being not comprehensible, thus, hard to work with. This conceptualization is similar to the definition of *cultural landscapes,* e.g., by UNESCO [89], in [70] or [71], where the latter also links to landscape conceptualization as area of distinct practices and a result of management choices as an adaptation to natural conditions (often traditional) or due to other reasons (subcategory 3.4). Though the complexity and diversity of cultural landscape definition is acknowledged here, this discussion is not in the scope of the current article. Relevant discussion can be found e.g., in [16,23,72].

In subcategory 3.3, the structure of agricultural landscapes has been characterised by Mottet et al. [73] as being made up of “agricultural terroirs” (or “landscape entities”), each with specific homogeneous characteristics in terms of natural environment, landscape planning and land use practices.

The studies covering conceptualizations within category 3 are addressing the different environmental and agricultural management perspectives of sustainable agriculture and its goals in the best way. These conceptualizations align with the ideas of Farina [72] of hierarchical landscape organisation with no specific spatial scale, and Sayer et al. [13] of landscapes defined by conceptual terms rather than by physical scales.

Meanwhile, in many cases it can be hard to set strict boundaries between the different (sub)categories. Instead, they may be seen within a continuum, where the accent shifts to different aspects (environmental, agronomic, social and cultural values), all relevant to agroecology and, thus, should not be excluded from a landscape definition.

From the overview of the publications (Table 1) it is noticeable that studies addressing similar topics often use different interpretations of landscape. However, the complexity of studies grows from the 1^st^ to the 3^rd^ category of conceptualization, which is supported by the increasing interdisciplinary aspects. More formal definitions - by size or other defined borders (categories 1 and 2) - are more characteristic for “classical” basic research (as defined in [74]) in environmental disciplines such as ecology or hydrology. On the other hand more holistic investigations with greater applicability potential view landscapes as complex and dynamic beings, acknowledging the significance of multifaceted human-nature relationships, which aligns with the ideas of Sayer et al. [13]. The authors outline specific examples of early landscape interpretations, such as in the context of exclusively visual characteristics, or of island-biogeography, or later ones, that acknowledge social-ecological relationships. They come to generically defining landscape as “an area delineated by an actor for a specific set of objectives. It constitutes an arena, in which entities, including humans, interact according to rules (physical, biological, and social) that determine their relationships” [13]. This definition may, however, be spatially restrictive and carries a risk of excluding important relevant processes, such as spill-over effects (e.g. [75]). The authors further describe *landscape approaches* as providing means for allocating and managing land for achieving social, economic, and environmental objectives in areas where different productive and environmental goals and respective land uses compete. They defined 10 principles supporting the interpretation of landscape approach, rather than providing its specific definition, which altogether highlights the importance of multiple objectives, stakeholder consideration and emphasize integration of agricultural and environmental priorities.

When landscape is defined by area size or border, the choice of where specifically to put the border is based on the balance between manageability and the ability to cover the functional aspects (in natural sciences). The terms, widely used in current literature with respect to scale, are “landscape approach” or “landscape perspective” (e.g. [56,76]), which roots to the question of the right scale of an investigation as a prerequisite to address the diversity of processes sufficiently. There is often a mismatch between ecological processes considered to be important and agricultural management [77] since ecological processes such as water and nutrient transport typically go beyond field or farm boundaries, while agricultural management is organised within the latter [78]. Moreover, for studying agroecosystems systematically, it becomes critical to account for cross-scale interactions [13,79]. In general, the increasing use of terms *landscape* or *landscape scale* in scientific literature highlights the urge to step away from smaller field or farm-scale research towards inclusion of larger number of interdependencies, often a subject of different disciplines. Sectoral approaches are increasingly recognized as unsuitable to satisfy the needs of such research [13]. Instead, a multi- and transdisciplinary vision needs to be accepted. Differing from disciplinary approaches, a common ground is not a matter of course and a common understanding of key terms needs to be developed. With respect to the topics to be integrated into the holistic research of agroecosystems, deciding on a unique spatial, temporal or organisational scale is realistic.

### 4.2 Landscape definition for research on sustainable agriculture

Here we conclude that landscape, as a system of interrelated both productive and non-productive habitats, can serve as a suitable unit for experimentation on management approaches to facilitate the redesign of agricultural production systems [56,80,81].

Based on integrating the above perspectives, the **definition of landscape** proposed here is as follows:

A **landscape** is a system of spatially arranged entities which are structurally and functionally interconnected.

This definition is not tied to geographical or temporal boundaries. Defined in such general terms, it allows for certain flexibility to consider the dynamic nature of relationships between environmental, economic, and often social elements of complex systems, such as agroecosystems. This enables continuous learning and adaptation processes for improved management in changing conditions and taking into account the unique local context.

An **a*gricultural landscape*** represents *a subset of landscape with high relevance of agricultural activities, including but not limited to arable fields, grassland and special crops.*

As mentioned by Mueller et al. [82], agricultural landscapes “are ‘cultural’ in a double sense”: the first is cultivation of crops and the other is rural culture with its values. Importantly, the above definition allows to grasp the connection to territory or place [7], which is necessary to avoid gaps in the innovation processes through research. This would also enable the consideration potential conflicts between natural and human-related processes, as highlighted by Farina [72] and the achievement of healthy multifunctional and resilient landscapes as seen through the lens of agroecology or sustainable intensification approaches [7].

In specific scenarios and pertaining to particular research topics, the definition of landscape and its spatial and temporal dimensions may become more concrete if the critical interrelations between these multiple dimensions of relevant agroecosystem features are identified. This way, for a specific research and its geographical location, the essential landscape elements can be identified, which will provide a more specific criteria for defining landscape in each case.

A common definition in such form will allow addressing systemic and social complexity of human-nature systems (as defined by Allain & Salliou [83]), such as in the interest of research on sustainable agriculture. This definition echoes the concept of integrated landscape management, as a tool for promoting sustainable agriculture in practice. Lack of actual integration between the disciplinary findings has been one of long-existing issues of many interdisciplinary projects in landscape research. This can be partly attributed to project design and coordination issues but is largely explained by the lack of common ground, including the understanding of terminology and the common goal [[83], [84], [85]]. In transdisciplinary research, the use of participatory methods allows to broaden our understanding of sustainability problems, but may result in a simplified and homogenized view of the landscape, hindering the recognition of ambiguities and innovative solutions to overcome challenges [83]. Responding to Palang's [86] comparison of landscape interpretations to the Indian story about blind men describing an elephant, we believe that our definition will allow to not only identify the “body parts”, but conclude with the full-body picture of an “elephant in motion”.

The practical application of this definition for a specific case would imply identifying the key interconnected entities at the first place. Especially in transdisciplinary projects, it can be useful to include open conversations with relevant actors as the first step, to get a higher-level idea in respect to practical goals and problem views. An example reflecting the importance of this step has been reported by Allain & Salliou [83], who used a Bayesian model constructed with local stakeholders to understand the disparity between landscape ecologists' recommendations and farmers' practices in pest control. The study demonstrated the divergence in understanding of relationships within the landscape system (referred as “technical incommensurability”) and supported the establishment of priorities of priorities in research for pest control. Sayer et al. [13] used workshops and consultations with multiple multidisciplinary experts to characterise landscape approach with 10 principles. The latter study also exemplifies the benefit of a shared (and rather general) definition of landscape (“an area delineated by an actor for a specific set of objectives”) as a starting point. Similarly, Sayer et al. [87] illustrated application of participatory approaches for developing a set of livehood and conservation indicators for simultaneous monitoring of conservation and development efforts, which also relied on building a shared understanding of landscape.

On a more technical level, the concretization of this definition, considering the complexity of real mixed socio-natural systems, requires a rethinking of the currently used monitoring and data analysis techniques. In particular, adoption of methods, which would allow to identify the links between the multiple features with the diversity of (non)linear and often unexpected relationships, will be essential. Such initiatives as on-farm or landscape experimentation in agriculture open the avenue towards this latter goal.

## 5 Conclusion

This article was motivated by the need to advance towards development of sustainable agricultural practices, which implies developing knowledge about functioning of complex systems through inter- and transdisciplinary research. Consensus on key concepts is a critical element for collaboration within respective projects, and scale of research is one of them. The discussions in the recent literature in this regard have increasingly focused on the concept of landscape, though its interpretation varies among the sources. This paper analysed a range of existing conceptualizations of landscape in relevant disciplines, which reflect different interpretations of “landscape”. The final objective was to arrive at a definition that could be commonly used and accepted by interdisciplinary scientists and practitioners. Three major categories of landscape conceptualizations with eight subcategories were identified. More formal spatial definitions of landscape are characteristic for classical and usually monodisciplinary research and were noticed in the minority of identified studies. An increasing number of studies emphasize the role of relationships between different components of the landscape in its interpretation, and are also characterised by growing interdisciplinarity of the corresponding studies.

We propose a definition of “landscape”, which can serve both as a “spatially abstract” scale for research in the agricultural context, including different levels of experimentation, and as a concept reflecting diversity of interrelated processes and phenomena considered research on sustainable agriculture. It leaves freedom to include specific aspects as modules depending on the research questions under study and complexity of a particular study.

To achieve systematic and applicable research for sustainable agriculture, we also face other challenges similar to those encountered in establishing agreement on a common research unit or scale. As a next step, a methodological foundation needs to be developed that enhances our comprehension of cause-effect relationships. This foundation will enable to explore synergies and trade-offs within the complex nature of real-world agricultural systems.

## Declaration of competing interest

The authors declare that they have no known competing financial interests or personal relationships that could have appeared to influence the work reported in this paper.
