# Supplementary material for: Use of the term “landscape” in sustainable agriculture research: A literature review
Source: Heliyon. 2023 Nov 10;9(11):e22173. doi: 10.1016/j.heliyon.2023.e22173 (PMC10694147; doi:10.1016/j.heliyon.2023.e22173)
Supplement: Multimedia component 1 [file mmc1.docx]

**Supplementary material**

1. R. Billeter, J. Liira, D. Bailey, R. Bugter, P. Arens, I. Augenstein, et al. Indicators for biodiversity in agricultural landscapes: a pan-European study. Journal of Applied Ecology. 2007;45:141–50. doi:10.1111/j.1365-2664.2007.01393.x.

2. Flavia Geiger, Jan Bengtsson, Frank Berendse, Wolfgang W. Weisser, Mark Emmerson, Manuel B. Morales, et al. Persistent negative effects of pesticides on biodiversity and biological control potential on European farmland. Basic and Applied Ecology. 2010;11:97–105. doi:10.1016/j.baae.2009.12.001.

3. Catrin Westphal, Ingolf Steffan-Dewenter, Teja Tscharntke. Mass flowering crops enhance pollinator densities at a landscape scale. Ecology Letters. 2003;6:961–5. doi:10.1046/j.1461-0248.2003.00523.x.

4. Jeff Holland, Lenore Fahrig. Effect of woody borders on insect density and diversity in crop fields: a landscape-scale analysis. Agriculture, Ecosystems & Environment. 2000;78:115–22. doi:10.1016/S0167-8809(99)00123-1.

5. Michale Glennon, William F. Porter. Using satellite imagery to assess landscape-scale habitat for wild turkeys. Wildlife Society Bulletin. 1999;27:646–53.

6. Timothy M. Bowles, Veronica Acosta-Martínez, Francisco Calderón, Louise E. Jackson. Soil enzyme activities, microbial communities, and carbon and nitrogen availability in organic agroecosystems across an intensively-managed agricultural landscape. Soil Biology and Biochemistry. 2014;68:252–62. doi:10.1016/j.soilbio.2013.10.004.

7. Leigh Winowiecki, Tor-Gunnar Vågen, Boniface Massawe, Nicolas A. Jelinski, Charles Lyamchai, George Sayula, Elizabeth Msoka. Landscape-scale variability of soil health indicators: effects of cultivation on soil organic carbon in the Usambara Mountains of Tanzania. Nutrient Cycling in Agroecosystems. 2016;105:263–74. doi:10.1007/s10705-015-9750-1.

8. Elizabeth W. Boyer, Christine L. Goodale, Norbert A. Jaworski, Robert W. Howarth. Anthropogenic nitrogen sources and relationships to riverine nitrogen export in the northeastern U.S.A. Biogeochemistry. 2002;57:137–69. doi:10.1023/A:1015709302073.

9. C Stoate, N.D Boatman, R.J Borralho, C.Rio Carvalho, G.R.de Snoo, P Eden. Ecological impacts of arable intensification in Europe. Journal of Environmental Management. 2001;63:337–65. doi:10.1006/jema.2001.0473.

10. C. Stoate, A. Báldi, P. Beja, N.D. Boatman, I. Herzon, A. van Doorn, et al. Ecological impacts of early 21st century agricultural change in Europe – A review. Journal of Environmental Management. 2009;91:22–46. doi:10.1016/j.jenvman.2009.07.005.

11. J. Sayer, T. Sunderland, J. Ghazoul, J.-L. Pfund, D. Sheil, E. Meijaard, et al. Ten principles for a landscape approach to reconciling agriculture, conservation, and other competing land uses. Proceedings of the National Academy of Sciences. 2013;110:8349–56. doi:10.1073/pnas.1210595110.

12. Paul H. Selman. Planning at the Landscape Scale: Routledge; 2006.

13. Monica G. Turner, Robert H. Gardner. Landscape Ecology in Theory and Practice: Springer New York; 2015.

14. M. Tveit, Å. Ode, G. Fry. Key concepts in a framework for analysing visual landscape character. Landscape Research. 2006;31:229–55. doi:10.1080/01426390600783269.

15. Nadja Penko Seidl, Mateja Šmid Hribar, Jelka Hudoklin, Tomaž Pipan, Mojca Golobič. Defining Landscapes, and Their Importance for National Identity—A Case Study from Slovenia. Sustainability. 2021;13:6475. doi:10.3390/su13116475.

16. D MacDonald, J.R Crabtree, G Wiesinger, T Dax, N Stamou, P Fleury, et al. Agricultural abandonment in mountain areas of Europe: Environmental consequences and policy response. Journal of Environmental Management. 2000;59:47–69. doi:10.1006/jema.1999.0335.

17. Nathalie van Vliet, Ole Mertz, Andreas Heinimann, Tobias Langanke, Unai Pascual, Birgit Schmook, et al. Trends, drivers and impacts of changes in swidden cultivation in tropical forest-agriculture frontiers: A global assessment. Global Environmental Change. 2012;22:418–29. doi:10.1016/j.gloenvcha.2011.10.009.

18. Zacharias Steinmetz, Claudia Wollmann, Miriam Schaefer, Christian Buchmann, Jan David, Josephine Tröger, et al. Plastic mulching in agriculture. Trading short-term agronomic benefits for long-term soil degradation? Science of The Total Environment. 2016;550:690–705. doi:10.1016/j.scitotenv.2016.01.153.

19. Margaret Buck Holland, Sierra Zaid Shamer, Pablo Imbach, Juan Carlos Zamora, Claudia Medellin Moreno, Efraín J. Leguía Hidalgo, et al. Mapping adaptive capacity and smallholder agriculture: applying expert knowledge at the landscape scale. Climatic Change. 2017;141:139–53. doi:10.1007/s10584-016-1810-2.

20. Alexandra-Maria Klein, Bernard E Vaissière, James H Cane, Ingolf Steffan-Dewenter, Saul A Cunningham, Claire Kremen, Teja Tscharntke. Importance of pollinators in changing landscapes for world crops. Proceedings of the Royal Society B: Biological Sciences. 2007;274:303–13. doi:10.1098/rspb.2006.3721.

21. Milton Cezar Ribeiro, Jean Paul Metzger, Alexandre Camargo Martensen, Flávio Jorge Ponzoni, Márcia Makiko Hirota. The Brazilian Atlantic Forest: How much is left, and how is the remaining forest distributed? Implications for conservation. Biological Conservation. 2009;142:1141–53. doi:10.1016/j.biocon.2009.02.021.

22. Caspar A. Hallmann, Martin Sorg, Eelke Jongejans, Henk Siepel, Nick Hofland, Heinz Schwan, et al. More than 75 percent decline over 27 years in total flying insect biomass in protected areas. PLOS ONE. 2017;12:e0185809. doi:10.1371/journal.pone.0185809.

23. F.J.J.A Bianchi, C.J.H Booij, T Tscharntke. Sustainable pest regulation in agricultural landscapes: a review on landscape composition, biodiversity and natural pest control. Proceedings of the Royal Society B: Biological Sciences. 2006;273:1715–27. doi:10.1098/rspb.2006.3530.

24. Teja Tscharntke, Jason M. Tylianakis, Tatyana A. Rand, Raphael K. Didham, Lenore Fahrig, Péter Batáry, et al. Landscape moderation of biodiversity patterns and processes - eight hypotheses. Biological Reviews. 2012;87:661–85. doi:10.1111/j.1469-185X.2011.00216.x.

25. Nicole E. Heller, Erika S. Zavaleta. Biodiversity management in the face of climate change: A review of 22 years of recommendations. Biological Conservation. 2009;142:14–32. doi:10.1016/j.biocon.2008.10.006.

26. Robert A. Robinson, William J. Sutherland. Post-war changes in arable farming and biodiversity in Great Britain. Journal of Applied Ecology. 2002;39:157–76. doi:10.1046/j.1365-2664.2002.00695.x.

27. Tscharntke T, Clough Y, Wanger TC, Jackson L, Motzke I, Perfecto I, et al. Global food security, biodiversity conservation and the future of agricultural intensification. Biological Conservation. 2012;151:53–9. doi:10.1016/j.biocon.2012.01.068.

28. Janne Bengtsson, Johan Ahnström, Ann-Christin Weibull. The effects of organic agriculture on biodiversity and abundance: a meta-analysis. Journal of Applied Ecology. 2005;42:261–9. doi:10.1111/j.1365-2664.2005.01005.x.

29. Lenore Fahrig, Jacques Baudry, Lluís Brotons, Françoise G. Burel, Thomas O. Crist, Robert J. Fuller, et al. Functional landscape heterogeneity and animal biodiversity in agricultural landscapes. Ecology Letters. 2011;14:101–12. doi:10.1111/j.1461-0248.2010.01559.x.

30. Claire Kremen, Neal M. Williams, Marcelo A. Aizen, Barbara Gemmill-Herren, Gretchen LeBuhn, Robert Minckley, et al. Pollination and other ecosystem services produced by mobile organisms: a conceptual framework for the effects of land-use change. Ecology Letters. 2007;10:299–314. doi:10.1111/j.1461-0248.2007.01018.x.

31. Thomas J. Stohlgren, Dan Binkley, Geneva W. Chong, Mohammed A. Kalkhan, Lisa D. Schell, Kelly A. Bull, et al. Exotic Plant Species Invade Hot Spots of Native Plant Diversity. Ecological Monographs. 1999;69:25. doi:10.2307/2657193.

32. Christina M. Kennedy, Eric Lonsdorf, Maile C. Neel, Neal M. Williams, Taylor H. Ricketts, Rachael Winfree, et al. A global quantitative synthesis of local and landscape effects on wild bee pollinators in agroecosystems. Ecology Letters. 2013;16:584–99. doi:10.1111/ele.12082.

33. Teja Tscharntke, Riccardo Bommarco, Yann Clough, Thomas O. Crist, David Kleijn, Tatyana A. Rand, et al. Conservation biological control and enemy diversity on a landscape scale. Biological Control. 2007;43:294–309. doi:10.1016/j.biocontrol.2007.08.006.

34. Simon G. Potts, Betsy Vulliamy, Amots Dafni, Gidi Ne’eman, Pat Willmer. LINKING BEES AND FLOWERS: HOW DO FLORAL COMMUNITIES STRUCTURE POLLINATOR COMMUNITIES? Ecology. 2003;84:2628–42. doi:10.1890/02-0136.

35. Neal M. Williams, Elizabeth E. Crone, T’ai H. Roulston, Robert L. Minckley, Laurence Packer, Simon G. Potts. Ecological and life-history traits predict bee species responses to environmental disturbances. Biological Conservation. 2010;143:2280–91. doi:10.1016/j.biocon.2010.03.024.

36. David Kleijn, Maj Rundlöf, Jeroen Scheper, Henrik G. Smith, Teja Tscharntke. Does conservation on farmland contribute to halting the biodiversity decline? Trends in Ecology & Evolution. 2011;26:474–81. doi:10.1016/j.tree.2011.05.009.

37. Péter Batáry, Lynn V. Dicks, David Kleijn, William J. Sutherland. The role of agri‐environment schemes in conservation and environmental management. Conservation Biology. 2015;29:1006–16. doi:10.1111/cobi.12536.

38. Frederik Hendrickx, JEAN-PIERRE MAELFAIT, WALTER VAN WINGERDEN, OLIVER SCHWEIGER, MARJAN SPEELMANS, Stéphanie Aviron, et al. How landscape structure, land-use intensity and habitat diversity affect components of total arthropod diversity in agricultural landscapes. Journal of Applied Ecology. 2007;44:340–51. doi:10.1111/j.1365-2664.2006.01270.x.

39. Taylor H. Ricketts. Tropical Forest Fragments Enhance Pollinator Activity in Nearby Coffee Crops. Conservation Biology. 2004;18:1262–71. doi:10.1111/j.1523-1739.2004.00227.x.

40. Teja Tscharntke, Yann Clough, Shonil A. Bhagwat, Damayanti Buchori, Heiko Faust, Dietrich Hertel, et al. Multifunctional shade-tree management in tropical agroforestry landscapes - a review. Journal of Applied Ecology. 2011;48:619–29. doi:10.1111/j.1365-2664.2010.01939.x.

41. Ann-Christin Weibull. Species richness in agroecosystems: the effect of landscape, habitat and farm management. Biodiversity and Conservation. 2003;12:1335–55. doi:10.1023/A:1023617117780.

42. Péter Batáry, András Báldi, David Kleijn, Teja Tscharntke. Landscape-moderated biodiversity effects of agri-environmental management: a meta-analysis. Proceedings of the Royal Society B: Biological Sciences. 2011;278:1894–902. doi:10.1098/rspb.2010.1923.

43. Andrew F. Bennett, James Q. Radford, Angie Haslem. Properties of land mosaics: Implications for nature conservation in agricultural environments. Biological Conservation. 2006;133:250–64. doi:10.1016/j.biocon.2006.06.008.

44. Sean L. Tuck, Camilla Winqvist, Flávia Mota, Johan Ahnström, Lindsay A. Turnbull, Janne Bengtsson. Land‐use intensity and the effects of organic farming on biodiversity: a hierarchical meta‐analysis. Journal of Applied Ecology. 2014;51:746–55. doi:10.1111/1365-2664.12219.

45. Renata Pardini, Adriana de Arruda Bueno, Toby A. Gardner, Paulo Inácio Prado, Jean Paul Metzger. Beyond the Fragmentation Threshold Hypothesis: Regime Shifts in Biodiversity Across Fragmented Landscapes. PLOS ONE. 2010;5:e13666. doi:10.1371/journal.pone.0013666.

46. Chazdon RL, Harvey CA, Komar O, Griffith DM, Ferguson BG, Martínez-Ramos M, et al. Beyond Reserves: A Research Agenda for Conserving Biodiversity in Human-modified Tropical Landscapes. Biotropica. 2009;41:142–53. doi:10.1111/j.1744-7429.2008.00471.x.

47. Tiziano Gomiero, David Pimentel, Maurizio G. Paoletti. Environmental Impact of Different Agricultural Management Practices: Conventional vs. Organic Agriculture. Critical Reviews in Plant Sciences. 2011;30:95–124. doi:10.1080/07352689.2011.554355.

48. Jens Dauber, Saori Miyake. To integrate or to segregate food crop and energy crop cultivation at the landscape scale? Perspectives on biodiversity conservation in agriculture in Europe. Energy, Sustainability and Society. 2016;6:25. doi:10.1186/s13705-016-0089-5.

49. Carl Stenoien, Kelly R. Nail, Jacinta M. Zalucki, Hazel Parry, Karen S. Oberhauser, Myron P. Zalucki. Monarchs in decline: a collateral landscape-level effect of modern agriculture. Insect Science. 2018;25:528–41. doi:10.1111/1744-7917.12404.

50. Annika L. Hass, Urs G. Kormann, Teja Tscharntke, Yann Clough, Aliette Bosem Baillod, Clélia Sirami, et al. Landscape configurational heterogeneity by small-scale agriculture, not crop diversity, maintains pollinators and plant reproduction in western Europe. Proceedings of the Royal Society B: Biological Sciences. 2018;285:20172242. doi:10.1098/rspb.2017.2242.

51. Patrick Zimmermann, Erich Tasser, Georg Leitinger, Ulrike Tappeiner. Effects of land-use and land-cover pattern on landscape-scale biodiversity in the European Alps. Agriculture, Ecosystems & Environment. 2010;139:13–22. doi:10.1016/j.agee.2010.06.010.

52. Elena Concepción, Mario Díaz. Relative effects of field-and landscape-scale intensification on farmland bird diversity in Mediterranean dry cereal croplands. Aspects of Applied Biology. 2010;100:2010.

53. Teja Tscharntke, Alexandra M. Klein, Andreas Kruess, Ingolf Steffan-Dewenter, Carsten Thies. Landscape perspectives on agricultural intensification and biodiversity â ecosystem service management. Ecology Letters. 2005;8:857–74. doi:10.1111/j.1461-0248.2005.00782.x.

54. Pierre Chopin, Jean-Marc Blazy, Thierry Doré. A new method to assess farming system evolution at the landscape scale. Agronomy for Sustainable Development. 2015;35:325–37. doi:10.1007/s13593-014-0250-5.

55. Megan E. O’Rourke, Matthew J. Petersen. Extending the ‘resource concentration hypothesis’ to the landscape-scale by considering dispersal mortality and fitness costs. Agriculture, Ecosystems & Environment. 2017;249:1–3. doi:10.1016/j.agee.2017.07.022.

56. P. Lavelle, T. Decaëns, M. Aubert, S. Barot, M. Blouin, F. Bureau, et al. Soil invertebrates and ecosystem services. European Journal of Soil Biology. 2006;42:S3-S15. doi:10.1016/j.ejsobi.2006.10.002.

57. Rong-Gang Cong, Henrik G. Smith, Ola Olsson, Mark Brady. Managing ecosystem services for agriculture: Will landscape-scale management pay? Ecological Economics. 2014;99:53–62. doi:10.1016/j.ecolecon.2014.01.007.

58. David J Abson, Evan DG Fraser, Tim G Benton. Landscape diversity and the resilience of agricultural returns: a portfolio analysis of land-use patterns and economic returns from lowland agriculture. Agriculture & Food Security. 2013;2:2. doi:10.1186/2048-7010-2-2.

59. Francisco Moreira, Olga Viedma, Margarita Arianoutsou, Thomas Curt, Nikos Koutsias, Eric Rigolot, et al. Landscape – wildfire interactions in southern Europe: Implications for landscape management. Journal of Environmental Management. 2011;92:2389–402. doi:10.1016/j.jenvman.2011.06.028.

60. Hugh Eva, Eric F. Lambin. Fires and land-cover change in the tropics:a remote sensing analysis at the landscape scale. Journal of Biogeography. 2000;27:765–76. doi:10.1046/j.1365-2699.2000.00441.x.

61. Elena M. Bennett, Garry D. Peterson, Line J. Gordon. Understanding relationships among multiple ecosystem services. Ecology Letters. 2009;12:1394–404. doi:10.1111/j.1461-0248.2009.01387.x.

62. Jules Pretty. Agricultural sustainability: concepts, principles and evidence. Philosophical Transactions of the Royal Society B: Biological Sciences. 2008;363:447–65. doi:10.1098/rstb.2007.2163.

63. Alexander Wezel, Marion Casagrande, Florian Celette, Jean-François Vian, Aurélie Ferrer, Joséphine Peigné. Agroecological practices for sustainable agriculture. A review. Agronomy for Sustainable Development. 2014;34:1–20. doi:10.1007/s13593-013-0180-7.

64. Gilles Lemaire, Alan Franzluebbers, Paulo César de Faccio Carvalho, Benoît Dedieu. Integrated crop–livestock systems: Strategies to achieve synergy between agricultural production and environmental quality. Agriculture, Ecosystems & Environment. 2014;190:4–8. doi:10.1016/j.agee.2013.08.009.

65. J.H.N. Palma, A.R. Graves, P.J. Burgess, K.J. Keesman, H. van Keulen, M. Mayus, et al. Methodological approach for the assessment of environmental effects of agroforestry at the landscape scale. Ecological Engineering. 2007;29:450–62. doi:10.1016/j.ecoleng.2006.09.016.

66. Adrian D. Manning, Joern Fischer, David B. Lindenmayer. Scattered trees are keystone structures – Implications for conservation. Biological Conservation. 2006;132:311–21. doi:10.1016/j.biocon.2006.04.023.

67. K.D. Holl, T.M. Aide. When and where to actively restore ecosystems? Forest Ecology and Management. 2011;261:1558–63. doi:10.1016/j.foreco.2010.07.004.

68. Vincent Colomb, Ophélie Touchemoulin, Louis Bockel, Jean-Luc Chotte, Sarah Martin, Marianne Tinlot, Martial Bernoux. Selection of appropriate calculators for landscape-scale greenhouse gas assessment for agriculture and forestry. Environmental Research Letters. 2013;8:15029. doi:10.1088/1748-9326/8/1/015029.

69. Dennis C. Duro, Steven E. Franklin, Monique G. Dubé. A comparison of pixel-based and object-based image analysis with selected machine learning algorithms for the classification of agricultural landscapes using SPOT-5 HRG imagery. Remote Sensing of Environment. 2012;118:259–72. doi:10.1016/j.rse.2011.11.020.

70. William L. Baker. A review of models of landscape change. Landscape Ecology. 1989;2:111–33. doi:10.1007/BF00137155.

71. Harini Nagendra, Richard Lucas, João Pradinho Honrado, Rob H.G. Jongman, Cristina Tarantino, Maria Adamo, Paola Mairota. Remote sensing for conservation monitoring: Assessing protected areas, habitat extent, habitat condition, species diversity, and threats. Ecological Indicators. 2013;33:45–59. doi:10.1016/j.ecolind.2012.09.014.

72. Richard T T Forman. Land mosaics : the ecology of landscapes and regions: Cambridge University Press; 1995.

73. Michael E. McClain, Elizabeth W. Boyer, C. Lisa Dent, Sarah E. Gergel, Nancy B. Grimm, Peter M. Groffman, et al. Biogeochemical Hot Spots and Hot Moments at the Interface of Terrestrial and Aquatic Ecosystems. Ecosystems. 2003;6:301–12. doi:10.1007/s10021-003-0161-9.

74. Klaus Butterbach-Bahl, Elizabeth M. Baggs, Michael Dannenmann, Ralf Kiese, Sophie Zechmeister-Boltenstern. Nitrous oxide emissions from soils: how well do we understand the processes and their controls? Philosophical Transactions of the Royal Society B: Biological Sciences. 2013;368:20130122. doi:10.1098/rstb.2013.0122.

75. Mary J Kraus. Paleosols in clastic sedimentary rocks: their geologic applications. Earth-Science Reviews. 1999;47:41–70. doi:10.1016/S0012-8252(99)00026-4.

76. J.M. Bowler. Aridity in Australia: Age, origins and expression in aeolian landforms and sediments. Earth-Science Reviews. 1976;12:279–310. doi:10.1016/0012-8252(76)90008-8.

77. Saskia Keesstra, Joao Nunes, Agata Novara, David Finger, David Avelar, Zahra Kalantari, Artemi Cerdà. The superior effect of nature based solutions in land management for enhancing ecosystem services. Science of The Total Environment. 2018;610-611:997–1009. doi:10.1016/j.scitotenv.2017.08.077.

78. Robert V. O’Neill, Carolyn T. Hunsaker, K. Bruce Jones, Kurt H. Riitters, James D. Wickham, Paul M. Schwartz, et al. Monitoring Environmental Quality at the Landscape Scale. BioScience. 1997;47:513–9. doi:10.2307/1313119.

79. Robert V. O’Neill, K. Bruce Jones, Kurt H. Riitters, Iris A. Goodman. LANDSCAPE MONITORING AND ASSESSMENT RESEARCH PLAN 1994.

80. R. Lal. Soil Erosion Impact on Agronomic Productivity and Environment Quality. Critical Reviews in Plant Sciences. 1998;17:319–464. doi:10.1080/07352689891304249.

81. Louise J. Bracken, Jacky Croke. The concept of hydrological connectivity and its contribution to understanding runoff-dominated geomorphic systems. Hydrological Processes. 2007;21:1749–63. doi:10.1002/hyp.6313.

82. PHILIP E. HULME. Beyond control: wider implications for the management of biological invasions. Journal of Applied Ecology. 2006;43:835–47. doi:10.1111/j.1365-2664.2006.01227.x.

83. J. M. Rey Benayas. Abandonment of agricultural land: an overview of drivers and consequences. CAB Reviews: Perspectives in Agriculture, Veterinary Science, Nutrition and Natural Resources 2007. doi:10.1079/PAVSNNR20072057.

84. Sara J Scherr, Jeffrey A McNeely. Biodiversity conservation and agricultural sustainability: towards a new paradigm of ‘ecoagriculture’ landscapes. Philosophical Transactions of the Royal Society B: Biological Sciences. 2008;363:477–94. doi:10.1098/rstb.2007.2165.

85. Joern Fischer, Berry Brosi, Gretchen C Daily, Paul R Ehrlich, Rebecca Goldman, Joshua Goldstein, et al. Should agricultural policies encourage land sparing or wildlife-friendly farming? Frontiers in Ecology and the Environment. 2008;6:380–5. doi:10.1890/070019.

86. Ralf-Uwe Syrbe, Ulrich Walz. Spatial indicators for the assessment of ecosystem services: Providing, benefiting and connecting areas and landscape metrics. Ecological Indicators. 2012;21:80–8. doi:10.1016/j.ecolind.2012.02.013.

87. Ingo Zasada. Multifunctional peri-urban agriculture—A review of societal demands and the provision of goods and services by farming. Land Use Policy. 2011;28:639–48. doi:10.1016/j.landusepol.2011.01.008.

88. Tobias Plieninger, Claudia Bieling. Resilience and the Cultural Landscape: Resilience and the Cultural Landscape: Understanding and Managing Change in human-shaped Environments: Cambridge University Press; 2012.

89. Peter Frost, Bruce Campbell, Gabriel Medina, Leonard Usongo. Landscape-scale Approaches for Integrated Natural Resource Management in Tropical Forest Landscapes. Ecology and Society. 2006;11:art30. doi:10.5751/ES-01932-110230.

90. Dawn C. Parker, Steven M. Manson, Marco A. Janssen, Matthew J. Hoffmann, Peter Deadman. Multi-Agent Systems for the Simulation of Land-Use and Land-Cover Change: A Review. Annals of the Association of American Geographers. 2003;93:314–37. doi:10.1111/1467-8306.9302004.

91. Serra P, Pons X, Saurí D. Land-cover and land-use change in a Mediterranean landscape: A spatial analysis of driving forces integrating biophysical and human factors. Applied Geography. 2008;28:189–209. doi:10.1016/j.apgeog.2008.02.001.

92. Anne Mottet, Sylvie Ladet, Nathalie Coqué, Annick Gibon. Agricultural land-use change and its drivers in mountain landscapes: A case study in the Pyrenees. Agriculture, Ecosystems & Environment. 2006;114:296–310. doi:10.1016/j.agee.2005.11.017.

93. Matthias Bürgi, Peter H. Verburg, Tobias Kuemmerle, Tobias Plieninger. Analyzing dynamics and values of cultural landscapes. Landscape Ecology. 2017;32:2077–81. doi:10.1007/s10980-017-0573-0.

94. Sara J Scherr, Seth Shames, Rachel Friedman. From climate-smart agriculture to climate-smart landscapes. Agriculture & Food Security. 2012;1:12. doi:10.1186/2048-7010-1-12.

95. Paul Caplat, Jacques Lepart, Pascal Marty. Landscape patterns and agriculture: modelling the long-term effects of human practices on Pinus sylvestris spatial dynamics (Causse Mejean, France). Landscape Ecology. 2006;21:657–70. doi:10.1007/s10980-005-4430-1.

96. Toby A. Gardner, Jos Barlow, Robin Chazdon, Robert M. Ewers, Celia A. Harvey, Carlos A. Peres, Navjot S. Sodhi. Prospects for tropical forest biodiversity in a human-modified world. Ecology Letters. 2009;12:561–82. doi:10.1111/j.1461-0248.2009.01294.x.

97. Val Plumwood. The Concept of a Cultural Landscape: Nature, Culture and Agency in the Land. Ethics and the Environment. 2006;11:115–50.

98. Lothar Mueller, Frank Eulenstein, Nikolai M. Dronin, Wilfried Mirschel, Blair M. McKenzie, Marc Antrop, et al. Agricultural Landscapes: History, Status and Challenges; 2021.

99. Lena Schaller, Stefano Targetti, Anastasio J. Villanueva, Ingo Zasada, Jochen Kantelhardt, Manuel Arriaza, et al. Agricultural landscapes, ecosystem services and regional competitiveness—Assessing drivers and mechanisms in nine European case study areas. Land Use Policy. 2018;76:735–45. doi:10.1016/j.landusepol.2018.03.001.

100. IUCN. 2019. Draft 2: Global Standard for Nature-based Solutions. Retrieved 17 January, 2022 from:<https://www.iucn.org/sites/dev/files/content/documents/2019/global_standard_for_nature-based_solutions_english.pdf>[.](https://www.iucn.org/sites/dev/files/content/documents/2019/global_standard_for_nature-based_solutions_english.pdf.)

101. UNESCO. Cultural Landscapes. Retrieved 20.01.2022 from <https://whc.unesco.org/en/culturallandscape/>.

102. Council of Europe 2000. European Landscape Convention (CETS no. 176). Retrieved 17 January 2022 from: <https://rm.coe.int/1680080621>

103. Alex Guenther, Chris Geron, Tom Pierce, Brian Lamb, Peter Harley, Ray Fall. Natural emissions of non-methane volatile organic compounds, carbon monoxide, and oxides of nitrogen from North America. Atmospheric Environment. 2000;34:2205–30. doi:10.1016/S1352-2310(99)00465-3.
